# Generalized pustular psoriasis in infant with heterozygous mutation in the IL36RN gene successfully treated with infliximab

**DOI:** 10.1186/1546-0096-12-S1-P79

**Published:** 2014-09-17

**Authors:** Mia Glerup, Jens Veirum, Lars Iversen, Mette Christiansen, Troels Herlin

**Affiliations:** 1 Pediatrics, Aarhus University Hospital, Skejby, Denmark; 2 Department of Dermatology, Aarhus University Hospital, Aarhus N, Denmark; 3 Clinical Immunology, Aarhus University Hospital, Aarhus N, Denmark

## Introduction

Homozygous missense mutation in the *IL36RN* gene resulting in deficiency of interleukin-36-receptor antagonist (DITRA) is phenotypically presented as severe generalized pustular psoriasis starting in early childhood. Compound heterozygous cases have been described with the same DITRA phenotype, but to our knowledge heterozygous *IL36RN* mutation related to severe generalized pustular psoriasis in early childhood has not been described.

## Objectives

Case report

## Methods

Case report

## Results

First child of non-consanguineous caucasian (Danish) parents prenatally diagnosed with tetralogy of Fallot. Array CGH revealed normal karyotype. Pregnancy and delivery was uneventful. Mother had hemorrhagic proctitis and psoriasis. The girl presented at 3 months of age with what appeared as infectious dermatitis and *S. aureus* cultured from skin lesions spreading to extremities and trunk. Blood tests including acute phase reactants were normal. She started on intravenous antibiotics and topical corticosteroids. During the following week the dermal changes presented with scaly sharply demarcated psoriasiform plaques. Infection was cleared and topical betamethasone gave a partial improvement. Cardiac surgery was performed at the age of 4 months. Procedures were uncomplicated but a precipitous flare of numerous pustules was then observed. Methotrexate treatment was initiated. On suspection for DIRA or DITRA genetic testing for *IL1RN* and *IL36RN* gene mutations was initiated. The girl was found to be heterozygous for a mutation in the *IL36RN* gene (exon 5, c 338C>T p Ser113Leu) whereas the *IL1RN* gene (mutated in DIRA patients) was normal. Additionally, a heterozygous mutation in the *NLRP3* gene was also found (exon 3, c.2107C>A, p.Gln703Lys) via whole exome sequencing. Treatment with anakinra (4 mg/kg/day) had a marked positive effect, but did not result in total remission. MTX was increased to 15 mg/m^2^/week given subcutaneously. After 8 weeks and optimized doses 8 mg/kg/day of anakinra without sufficient remission the treatment was shifted to infliximab 6.5 mg/kg/dose on Day 0, 14, 28, hereafter every 4 weeks with excellent effect within few days on skin, general condition and thrive.

## Conclusion

To the best of our knowledge we report the first detailed description of an infant with heterozygous S113L *IL36Ra* mutation along with heterozygous Q705K *NLRP3* mutation, phenotypically expressed as DITRA with severe generalized pustular psoriasis. Reduction of the IL36Ra function will lead to excessive activity of cytokines belonging to the IL-1 family, furthermore the gain-of-function mutation in NLRP3 will lead to excessive IL-1b and IL-18 production. Collectively, it makes it conceivable that anti-IL-1 treatment would exert an effect on the disease. Accordingly anakinra has previously been reported with a successful result for the treatment of DITRA (1). Also in our patient anakinra showed a marked effect on general condition, reduced the eruptions of pustular lesions, but the erythrodermal changes were preserved and therefore only a partial response of the skin lesions could be registered. However, we demonstrate in this infantile DITRA patient that TNF-alpha inhibition with infliximab dramatically improved the dermal changes and could normalize the skin within few weeks.

## Disclosure of interest

None declared.

